# Supplementary material for: Two functionally distinct amyloid-β intermediates link aggregation pathways to pathology in Alzheimer’s disease
Source: Nat Commun. 2026 Nov 9;17:11333. doi: 10.1038/s41467-026-79028-0 (PMC13748623; doi:10.1038/s41467-026-79028-0)
Supplement: Supplementary file 2 [file 41467_2026_79028_MOESM2_ESM.pdf]

nature portfolio

Corresponding author(s): Georg Meisl, Jan Johason, Suman De

Last updated by author(s): Jun 19, 2026

Reporting Summary

Nature Portfolio wishes to improve the reproducibility of the work that we publish. This form provides structure for consistency and transparency in reporting. For further information on Nature Portfolio policies, see our Editorial Policies and the Editorial Policy Checklist.

Statistics

For all statistical analyses, confirm that the following items are present in the figure legend, table legend, main text, or Methods section.

n/a	Confirmed	
☐	☒	The exact sample size (*n*) for each experimental group/condition, given as a discrete number and unit of measurement
☐	☒	A statement on whether measurements were taken from distinct samples or whether the same sample was measured repeatedly
☐	☒	The statistical test(s) used AND whether they are one- or two-sided<br>*Only common tests should be described solely by name; describe more complex techniques in the Methods section.*
☐	☒	A description of all covariates tested
☒	☐	A description of any assumptions or corrections, such as tests of normality and adjustment for multiple comparisons
☐	☒	A full description of the statistical parameters including central tendency (e.g. means) or other basic estimates (e.g. regression coefficient) AND variation (e.g. standard deviation) or associated estimates of uncertainty (e.g. confidence intervals)
☐	☒	For null hypothesis testing, the test statistic (e.g. *F*, *t*, *r*) with confidence intervals, effect sizes, degrees of freedom and *P* value noted<br>*Give P values as exact values whenever suitable.*
☒	☐	For Bayesian analysis, information on the choice of priors and Markov chain Monte Carlo settings
☒	☐	For hierarchical and complex designs, identification of the appropriate level for tests and full reporting of outcomes
☒	☐	Estimates of effect sizes (e.g. Cohen's *d*, Pearson's *r*), indicating how they were calculated

Our web collection on statistics for biologists contains articles on many of the points above.

Software and code

Policy information about availability of computer code

Data collection	No custom code was developed for this work. Acquisition of single-aggregate imaging data was performed using Micro-Manager 2.0, while cell imaging data were acquired with the PerkinElmer Opera Phenix High Content Screening system and Incucyte live cell imaging. Protein aggregation, and ELISA measurements were obtained using a BMG-Labtech plate reader. MSD analyses were carried out on a MESO QuickPlex SQ 120 multiplex imager. Single-aggregate imaging and membrane permeabilization assays were performed using a home-built TIRF microscope based on a Nikon Ti2 body. Further details are provided in the Methods section.
Data analysis	Data analysis was performed using standard, widely available software packages. Cell imaging data were analyzed using PerkinElmer Harmony Analysis software and neurite length measurement were performed using IncuCyte® NeuroTrack Analysis Module. Kinetic fitting of protein aggregation data was carried out using AmyloFit based program. Imaging data were further processed in Fiji (ImageJ). Plate reader and MSD data were analyzed using manufacturer-provided software. Data analysis and plotting were performed in Origin 9.0, and final figures were prepared using Adobe Illustrator CS6. Statistical analyses were conducted in origin 9.0 , as detailed in the Methods section. No custom analysis code was developed for this study.

For manuscripts utilizing custom algorithms or software that are central to the research but not yet described in published literature, software must be made available to editors and reviewers. We strongly encourage code deposition in a community repository (e.g. GitHub). See the Nature Portfolio guidelines for submitting code & software for further information.

Data

Policy information about availability of data

All manuscripts must include a data availability statement. This statement should provide the following information, where applicable:

- Accession codes, unique identifiers, or web links for publicly available datasets
- A description of any restrictions on data availability
- For clinical datasets or third party data, please ensure that the statement adheres to our policy

All data made publicly available upon publication. All the analysed data will bw deposited in a spreadsheet with the manuscript

Human research participants

Policy information about studies involving human research participants and Sex and Gender in Research.

Reporting on sex and gender	Gender information for all patients and control can be found on the method section. t. Human postmortem AD tissue included samples from two female donors and one male donor. Human iPSC-derived neurons were generated from the male donor-derived KOLF2.1J iPSC line. Human iAstrocytes were generated from fibroblasts from a healthy female donor. Sex information was obtained from tissue-provider, sample-provider or cell-line metadata. Gender information was not available. Sex-stratified analyses were not performed because the study was not designed or powered to assess sex-specific effects.
Population characteristics	We included brain tissue samples from 3 individuals diagnosed with Alzheimer's disease. The Alzheimer's disease cases were neuropathologically confirmed. We used one human iPSC cortical neuron derived from KOLF2.1J (JIPSC001162) and one iAstrocyte lines neurologically healthy control, respectively.
Recruitment	No participants were recruited de novo for this study. The work was performed using existing human postmortem tissue samples and previously established human cell lines/cell-derived models. All samples and cell lines were obtained from approved sources and used in accordance with the relevant ethical approvals, consent procedures and institutional guidelines.
Ethics oversight	Postmortem human brain tissue was obtained from the Sheffield Brain Tissue Bank (SBTB), University of Sheffield, which operates under ethical approval as a Research Tissue Bank from the Wales Research Ethics Committee 5 (Reference 24/WA/0052). Informed consent was obtained from all donors during life or from their relatives. Fibroblasts used to generate iAstrocytes were obtained through the MODEL-AD research study under ethical approval from the Yorkshire & The Humber Research Ethics Committee (Reference 16/YH/0155), with informed consent obtained from all participants.KOLF2.1J-derived iPSC lines were obtained from The Jackson Laboratory/iPSC Neurodegenerative Disease Initiative resource and used in accordance with the supplier’s usage terms and conditions.

Note that full information on the approval of the study protocol must also be provided in the manuscript.

Field-specific reporting

Please select the one below that is the best fit for your research. If you are not sure, read the appropriate sections before making your selection.

☒ Life sciences ☐ Behavioural & social sciences ☐ Ecological, evolutionary & environmental sciences

For a reference copy of the document with all sections, see nature.com/documents/nr-reporting-summary-flat.pdf

Life sciences study design

All studies must disclose on these points even when the disclosure is negative.

Sample size	No statistical methods were used to pre-determine sample sizes
Data exclusions	No data were excluded from the analysis. The results of all the replicates are consistently similar to each other.
Replication	All experiments were performed with a minimum of three biological replicates. The exact number of replicates for each experiment is provided in the corresponding figure captions.
Randomization	All imaging data, including protein-aggregate imaging, membrane permeabilization assays, and cell imaging, were collected using automated microscope stage movement to eliminate potential user bias. For the other types of experiments in this study, randomization was not applicable.
Blinding	Blinding was not relevant to our study.

Reporting for specific materials, systems and methods

We require information from authors about some types of materials, experimental systems and methods used in many studies. Here, indicate whether each material, system or method listed is relevant to your study. If you are not sure if a list item applies to your research, read the appropriate section before selecting a response.

Materials & experimental systems

n/a	Involved in the study
☐	☒ Antibodies
☐	☒ Eukaryotic cell lines
☒	☐ Palaeontology and archaeology
☒	☐ Animals and other organisms
☒	☐ Clinical data
☒	☐ Dual use research of concern

Methods

n/a	Involved in the study
☒	☐ ChIP-seq
☒	☐ Flow cytometry
☒	☐ MRI-based neuroimaging

Antibodies

Antibodies used

Following Primary antibodies were used- .
Anti-Aβ40 (EPR23712-2, Abcam)
Anti-Aβ42 (21F12, Absolute Antibody), Ab02391-23.0
Alexa Fluor 647–labeled 21F12 antibody (Labelling performed in house)
Anti-pyroglutamate antibody (Abcam, IBR-10-2-3)
Isotype control antibody (ThermoFisher, 02-6300)
Anti-Nanog (Cell Signaling, 4903)
Anti-Pax6 (BioLegend, 901302)
Anti-FoxG1 (Invitrogen, PA5-2679)
Anti-Ctip2 (Abcam, ab18465)
Anti-βIII-tubulin (Abcam, ab18207)
Anti-MAP2 (Abcam, ab5392).
Anti-vimentin (Merck Millipore, AB5733)
Anti-CD44 (Abcam, ab157107)
Anti-EAAT2 (Abcam, ab41621),
Anti-Aβ (6E10; BioLegend, 803001)

Following Secondary antibodies were used- .
Goat anti-Rabbit IgG (H+L) Cross-Adsorbed Alexa Fluor™488 ThermoFisher A-11008 (Tissue -IHC)
Goat anti-Mouse IgG (H+L) Cross-Adsorbed, Alexa Fluor™47 ThermoFisher A-11004 (Tissue IHC)
Goat anti-Mouse IgG (H+L) Highly Cross-Adsorbed, Alexa Fluor™ 488 ThermoFisher, A-11029 (Cell- IC)
Donkey anti-Rabbit IgG (H+L) Highly Cross-Adsorbed , Alexa Fluor™ 647 ThermoFisher, A-31573 (Cell -IC)
Goat anti-Chicken IgY (H+L) Secondary Antibody, Alexa Fluor™ 488 ThermoFisher, A-11039 (Cell -IC)
Donkey anti-Rabbit IgG (H+L) Highly Cross-Adsorbed, Alexa Fluor™ 488 ThermoFisher, A-21206; (Cell -IC)

Antibody conjugation kit
Alexa Fluor™ 647 Conjugation Kit - Lightning-Link® ab269823
Alexa Fluor™ 488 Conjugation Kit - Lightning-Link® ab236553

Validation

All the antibodies used in this work are validated by the manufacturer and cited multiple times.

Eukaryotic cell lines

Policy information about cell lines and Sex and Gender in Research

Cell line source(s)	Human fibroblast lines used to generate human astrocyte like cell lines were obtained funder MTA. Lines derived from neurologically healthy individuals collected through forearm skin biopsies performed at the University of Sheffield.
Authentication	All cell lines were validated by immunocytochemistry using well-established markers and have also been previously reported in publications.
Mycoplasma contamination	All lines were regularly tested and confirmed to be negative for mycoplasma using the Mycoplasma Detection Kit
Commonly misidentified lines (See ICLAC register)	No commonly misidentified lines were used for this study.
