# Supplementary material for: Two functionally distinct amyloid-β intermediates link aggregation pathways to pathology in Alzheimer’s disease
Source: Nat Commun. 2026 Nov 9;17:11333. doi: 10.1038/s41467-026-79028-0 (PMC13748623; doi:10.1038/s41467-026-79028-0)
Supplement: Supplementary file 3 [file 41467_2026_79028_MOESM3_ESM.pdf]

nature portfolio

Peer Review File

Two functionally distinct amyloid-β intermediates link aggregation pathways to pathology in Alzheimer's disease

Corresponding Author: Dr Suman De

This manuscript has been previously reviewed at another journal. This document only contains information relating to versions considered at Nature Communications.

This file contains all reviewer reports in order by version, followed by all author rebuttals in order by version.

Version 0:

Reviewer comments:

Reviewer #3

(Remarks to the Author)
I am pleased to see that some concerns have been addressed in a reasonable manner upon the transfer of manuscript to Nature Communications. However, critical issues remain. First, I am inclined to agree with the other two reviewers: the main technical issue is that the chemical compositions of these two early Aβ1-42 aggregates stand as two black boxes. Moreover, the presumption behind the data comparison cannot stand that the on-pathway and off-pathway dynamics remain unchanged in the presence and absence of an exotic molecule. Together, the novelty and significance are compromised significantly and cannot meet the journal's scope because both the on-pathway and off-pathway oligomers have been reported to be cytotoxic species.

Major comments:

1. The authors must characterize the chemical compositions of these two different kinetic intermediates at least in the biophysical analysis in vitro. The super-resolution imaging and the sucrose gradient fractionation are two good starting points, but the results are not sufficient to rationalize the main findings of this work for their significantly different member-disrupting abilities in both in vitro and in vivo models.

2. To my knowledge, the rationale to use the half-time of the ThT fluorescence signals to normalize different Aβ aggregate samples is to have the same level of cross-β interactions. It holds true if there are only minor Aβ1-42 concentration variations (between 2 μM and 5 μM) because the authors could presume that there was no or little influence on both off-pathway and on-pathway aggregation dynamics. However, after adding 10% and 100% molecular equivalents of BRICHOS R221E, the on-pathway and off-pathway aggregation dynamics could no longer be presumed to be influenced insignificantly even at the same Aβ1-42 concentration (3 μM). In Figure 2D, no BRICHOS R221E-treated sample was analyzed at 85 minutes while 10% BRICHOS R221E-treated sample was analyzed at 105 minutes and 100% BRICHOS R221E-treated sample was analyzed at 275 minutes. A precondition is needed to carry out data comparison: relative to the no BRICHOS R221E-treated control, both on-pathway and off-pathway aggregation dynamics were only slowed down proportionately upon the BRICHOS R221E addition. An unverified presumption stood here that no significant aggregation pathway modulations and redirections took place. I strongly disagree. When BRICHOS R221E is involved, the authors must rule out its possible influence on the aggregation dynamics before normalizing to the 50% of the ThT fluorescence signals when incubated with 3 μM Aβ1-42. As an alternative option, the authors can choose to use the same time point when the on-pathway and off-pathway dynamics was the only variation. In other words, experimental data cannot be compared in a canonical manner when two variations may be activated.

3. The authors stated that BRICHOS R221E was a chaperone known to slow down aggregation by specifically inhibiting secondary nucleation. In contrast, as the authors discussed on page 17 and showed in Figure 8, both Class A and Class B arised predominantly from primary nucleation. These results seem to stand against each other. If BRICHOS R221E specifically inhibits secondary nucleation, the experiment results should show that only Class B is inhibited in the late aggregation time while no or little inhibiting effects should be observed in the lag phase where the primary nucleation predominates. The experimental results in this work show that BRICHOS R221E can inhibit both Class A and Class B

species. Being faithful to the results, I would rather deduce that the peptide-peptide interface(s), where BRICHOS R221E can act, may play a more important role in the formation of Class A species than that of Class B species. This also echoes well to the sucrose gradient fraction analysis: “lower-density fractions were enriched in class A species, whereas higher-density fractions were enriched in class B aggregates.” This hints that the authors may need to characterize the conformational differences between Class A and Class B in case the two species showed similar chemical compositions. I recommend that the authors could use known small-molecule amyloid inhibitors rather than BRICHOS R221E to explore the compositional and conformational differences between these two kinetic intermediates.

4. The authors extracted diffusible Aβ1-42 aggregates from the PM frontal cortex tissue of three AD patients. This means that these diffusible Aβ1-42 aggregates by large derives from the amyloid plaques. However, the authors need to be aware that these diffusible Aβ1-42 aggregates may have been chemically engineered upon the amyloid plaque formation. For example, cross-linked amyloid-β dimers have been reported to neurotoxic species such as the Asp1-Glu22 link (Brinkmalm et al., Brain 2019, 142, 1441).

Minor comments:

5. In Figure 4 and Figure 8, the on-pathway and off-pathway aggregations should be noted as reversible processes.

6. The legend to Figure 4 is a bit messy.

Reviewer #4

(Remarks to the Author)
The authors have gone to great lengths to address the concerns presented to them from all reviewers.

The data that has now been included in the manuscript and the SI adds strength to the study and removes doubt that aggregates were subject to membrane-associated, calcium buffer or dilution effects. The addition of the control experiment assessing BRICHOS affect on vesicle integrity in the absence of Abeta also strengthens the manuscript. The quantification of aggregates in the different brain regions adds clarity for the reader. The use of additional orthogonal biophysical characterization techniques is also beneficial to get a sense of the biophysical properties of these Class A and Class B aggregates.

In light of these additional data and modifications to the manuscript, I recommend this paper for publication with no further comments.

Reviewer #5

(Remarks to the Author)
Urbanek et al perform a series of studies to show the correlation of two functionally distinct amyloid-beta (Ab) intermediates with the pathology of Alzheimer’s disease.

Study 1: A single-vesicle assay is developed to quantify the membrane-disrupting activity of Ab42 aggregates. Specifically, the nanoscale vesicles composed of neuronal membrane-mimicking lipids were loaded with a dextran-conjugated Ca2+-sensitive dye (Cal-520). The vesicles were then immobilized in a Ca2+-containing buffer on a coverslip and exposed to Ab42 aggregates obtained at different time points in the aggregation kinetics (based on the ThT time trace). The disruption of vesicle membrane was measured by recording Ca2+ influx events. The two peaks in the distribution of these Ca2+ influx events are assumed to associate with two different classes of Ab42 intermediates: Class A intermediates with Ca2+ influx distribution peaking at ~ 5 Ca2+ ions per event and Class B intermediates with Ca2+ influx distribution peaking at ~ 2-3 Ca2+ ions per event. In the same set of experiments, the authors show that BRICHOS R221E (a chaperone known to slow down aggregation by specifically inhibiting secondary nucleation), with concentration equal to that of the Ab42 monomers, completely eliminated the Ca2+ influx events corresponding to Class A events, where as Ca2+ events associated with Class B intermediates remained detectable. They further showed that Ab42 significantly impaired neurite outgrowth in hPSC cortical neurons, which was restored to almost control levels when treated with BRICHOS R221E having the same concentration as that of Ab42.

Considering that the classification is based on the Ca2+ influx (functional) and not the extent of physical disruption of the membrane, this set of experiments support the existence of two predominant classes of functional Ab42 intermediates as claimed. I have only one minor concern about this study. No attempt has been made to explain or hypothesize the potential mechanism for the membrane disruption. Based on Figure 1I and 1J, the first Ca2+ influx event seems to occur in the first 1-3 minutes of exposing the vesicles to Ab42 intermediates. What would be the possible mechanism for such rapid membrane disruption?

Study 2: The next set of experiments show that the two distinct Ab42 intermediate classes have distinct kinetics signatures and damaging potential. This is shown by exposing the vesicles to Ab42 aggregates obtained at different time points along the aggregation kinetics (based of ThT signal) and plotting the distribution Ca2+ influx events. At earlier time, these distributions show a dominant peak corresponding to Class A events, which slowly decays with the time along the aggregation kinetics. The peak corresponding to Class B events, on the other hand, becomes pronounced and persists for the remaining of aggregation kinetics. The second part of this study uses a kinetics model to globally fit the ThT traces and

show that the model results in a better fit when Class A intermediates are considered to grow along the off-pathway and Class B intermediates are considered to grow along the on-pathway (seeds for the fibrils).

The experiments presented support the claim that the two distinct Ab42 intermediate classes have different damaging potentials, with Class A intermediates more toxic than Class B intermediates. However, the claim about the distinct kinetics has a caveat that needs to be acknowledged. After transferring the intermediates to the vesicles, the aggregate species are assumed to keep the same kinetic state (e.g. species that were off-pathway in the solution remain off-pathway after getting transferred to the vesicles) for the duration of 20 min (or longer) experiment.

Function 2F1 in equation 2 is not defined.

Study 3: Based on immunohistochemistry of frontal cortex from three AD cases, this set of experiments show that human AD brain contains two classes of toxic Ab42 intermediates, and extracts from these AD-affected brains impair neurite outgrowth in hPSC-derived cortical neurons.

The conclusions drawn from these experiments are well-supported by the data.

Study 4: This set of experiments show that BRICHOS R221E suppress the formation of Ab42 oligomeric intermediates and attenuates neuronal and astrocytic dysfunction. This includes restoration of neurite outgrowth in neurons and reduction in pro-inflammatory cytokines.

The conclusions drawn from these experiments are well-supported by the data.

Study 5: The two toxic Ab42 intermediates are also detected in a mouse model of AD, and BRICHOS R221E specifically suppress Class A intermediates in this mouse model.

The conclusions drawn from these experiments are well-supported by the data.

General comments:

1. Functional classification without direct structural characterization: While the authors classify the two intermediate classes based on their Ca^{2+} influx signatures, the structural basis for this functional distinction remains indirect. The sucrose gradient fractionation and dSTORM imaging data (Fig. S7) provide supporting evidence for physical distinctness between Class A and Class B species, but a more direct structural characterization — such as native mass spectrometry or cryo-EM of isolated fractions — would strengthen the claim that these functional classes correspond to structurally distinct assemblies. The authors should acknowledge this as a limitation of the current classification approach.

2. Lipid vesicle composition versus native neuronal membranes: The membrane permeabilization assay employs vesicles composed of neuronal membrane-mimicking lipids, which, while carefully chosen, do not fully recapitulate the complexity of native neuronal membranes. Factors such as membrane asymmetry, protein composition, cholesterol content, and membrane tension in living cells could influence the Ca^{2+} influx signatures and the relative membrane-disrupting potency of Class A and Class B intermediates. It would be valuable for the authors to discuss whether the functional classification and the relative potency of the two intermediate classes are expected to hold under more physiologically relevant membrane conditions.

3. Limited human brain cohort with unexplained inter-individual variability: The demonstration that both Class A and Class B intermediates are present in human Alzheimer's brain is an important finding; however, it is based on postmortem frontal cortex tissue from only three sporadic AD cases. Furthermore, the relative contributions of Class A and Class B intermediates vary considerably across the three patients (Fig. 5F), yet this variability is not discussed. It remains unclear whether these differences reflect disease stage, rate of progression, individual genetic background, or other factors. The authors should acknowledge the limited sample size as a constraint on the generalizability of their conclusions and discuss possible explanations for the observed inter-individual variability in intermediate class distribution.

4. Kinetic modeling assumptions underlying the on- and off-pathway classification: The assignment of Class A intermediates as off-pathway and Class B intermediates as on-pathway to fibril formation rests substantially on global kinetic modeling rather than direct experimental demonstration of interconversion or incorporation into fibrils. While the modeling approach is rigorous and the differential sensitivity to BRICHOS R221E provides complementary support, the conclusions are inherently dependent on the assumptions built into the kinetic framework — in particular, the reaction orders and the relative contributions of primary versus secondary nucleation. The authors should more explicitly discuss the limitations of this modeling approach and clarify how sensitive their pathway assignments are to variations in these underlying assumptions.

Overall: I find this study very interesting and significant for further progress in the field. The authors have done a very good job in responding to the previous round of reviews. I recommend the publication of this paper in Nature Communications after the concerns raised above are addressed.

Version 1:

Reviewer comments:

Reviewer #3

(Remarks to the Author)
I have a careful go on the main text and the rebuttal letter. I am pleased to say that the authors have adequately addressed the points that I raised in the last-round peer review. With additional results, the structural differences between class A and B have been clarified better (though the two species have yet to be characterized), the origins of class A and B are explained more straightforwardly, and the mechanism of action of BRICHOS R221 E is studied more thoroughly. Overall, I recommend publishing this manuscript as it stands.

Reviewer #5

(Remarks to the Author)
The authors have addressed all of my concerns. I have no further comments. I recommend this paper for publication in Nature Communications.

Open Access This Peer Review File is licensed under a Creative Commons Attribution 4.0 International License, which permits use, sharing, adaptation, distribution and reproduction in any medium or format, as long as you give appropriate credit to the original author(s) and the source, provide a link to the Creative Commons license, and indicate if changes were made.
In cases where reviewers are anonymous, credit should be given to 'Anonymous Referee' and the source.
The images or other third party material in this Peer Review File are included in the article's Creative Commons license, unless indicated otherwise in a credit line to the material. If material is not included in the article's Creative Commons license and your intended use is not permitted by statutory regulation or exceeds the permitted use, you will need to obtain permission directly from the copyright holder.
To view a copy of this license, visit https://creativecommons.org/licenses/by/4.0/

We thank the reviewers for their thorough assessment of our manuscript. Their comments have helped us to make the paper clearer, more robust, and more grounded in its mechanistic interpretation. We have addressed each point raised in a point-by-point manner below. (reviewer comments in blue, our responses in black, and text additions to the manuscript in green, references are hyperlinked for convenience).

Reviewer #3 (Remarks to the Author):

I am pleased to see that some concerns have been addressed in a reasonable manner upon the transfer of manuscript to Nature Communications. However, critical issues remain. First, I am inclined to agree with the other two reviewers: the main technical issue is that the chemical compositions of these two early Aβ1-42 aggregates stand as two black boxes. Moreover, the presumption behind the data comparison cannot stand that the on-pathway and off-pathway dynamics remain unchanged in the presence and absence of an exotic molecule. Together, the novelty and significance are compromised significantly and cannot meet the journal's scope because both the on-pathway and off-pathway oligomers have been reported to be cytotoxic species.

Reply: We thank the reviewer for their assessment of our manuscript. We have performed additional experiments, which we believe directly address the issues raised by the reviewer.

Major comments:

1. The authors must characterize the chemical compositions of these two different kinetic intermediates at least in the biophysical analysis in vitro. The super-resolution imaging and the sucrose gradient fractionation are two good starting points, but the results are not sufficient to rationalize the main findings of this work for their significantly different member-disrupting abilities in both in vitro and in vivo models.

Reply: We would like to clarify that the central aim of this study is not to provide high-resolution structural characterization of all Aβ intermediates, but the objective is to establish a functional assay capable of detecting individual Aβ aggregates based on disease-relevant membrane-disrupting activity, and to determine whether species with different damaging capacities arise through distinct aggregation routes. Using this approach, we resolve subpopulations of Aβ intermediates with different damaging activities at the single-aggregate level and define the pathways through which they form. We further traced their functional signatures across recombinant Aβ42, Alzheimer's disease mouse models, and human Alzheimer's brain, demonstrating both the broad applicability of the assay and the biological relevance of the detected species. The ability to resolve rare, transient, functionally distinct intermediates across disease-associated models and relate them to their routes of formation represents a key advance that is difficult to achieve with existing conventional methodologies.

A comprehensive structural analysis of all intermediates lies outside the scope of this kinetics- and method-development-focused study. Such investigations will be valuable in future work, but including them here would shift the focus away from the primary goals of establishing the assay and defining the functional and mechanistic distinctions between intermediate classes.

Nonetheless, we have now performed additional experiments as requested. Because both Class A and Class B intermediates arise from the same recombinant Aβ42 monomer in the same aggregation reaction, we do not expect differences in peptide composition per se. For this reason, our focus was on identifying physicochemical properties that could explain their distinct membrane-disrupting activities, rather than on chemical-composition differences that are not expected for intermediates derived from the same peptide pool.

First, we performed sucrose-gradient fractionation to enrich distinct aggregate populations, followed by ANS fluorescence measurements to assess surface hydrophobicity (**Fig. 1**). The membrane-disrupting profiles differed markedly across fractions: the 10-20% fraction, enriched in Class A species, showed Ca^{2+} influx distributions shifted toward higher-amplitude events, whereas the 30-40% fraction, enriched in Class B species, showed lower-amplitude distributions **(Fig. 1F)**. ANS fluorescence further revealed a blue-shifted emission spectrum in the 10-20% fraction compared with the 30-40% fraction (**Fig. 1H**), indicating greater exposed hydrophobicity in the Class A-enriched population. Because hydrophobic exposure is a key determinant of lipid membrane permeabilization (***Chem. Sci.*** *2019,10,6072*; ***PNAS*** *2016,113,10866*) these data support the idea that the higher surface hydrophobicity of Class A intermediates contributes to their stronger membrane-disrupting activity. We have updated the supplementary figure as Fig. S7 and revised the manuscript accordingly (Line 264-272).

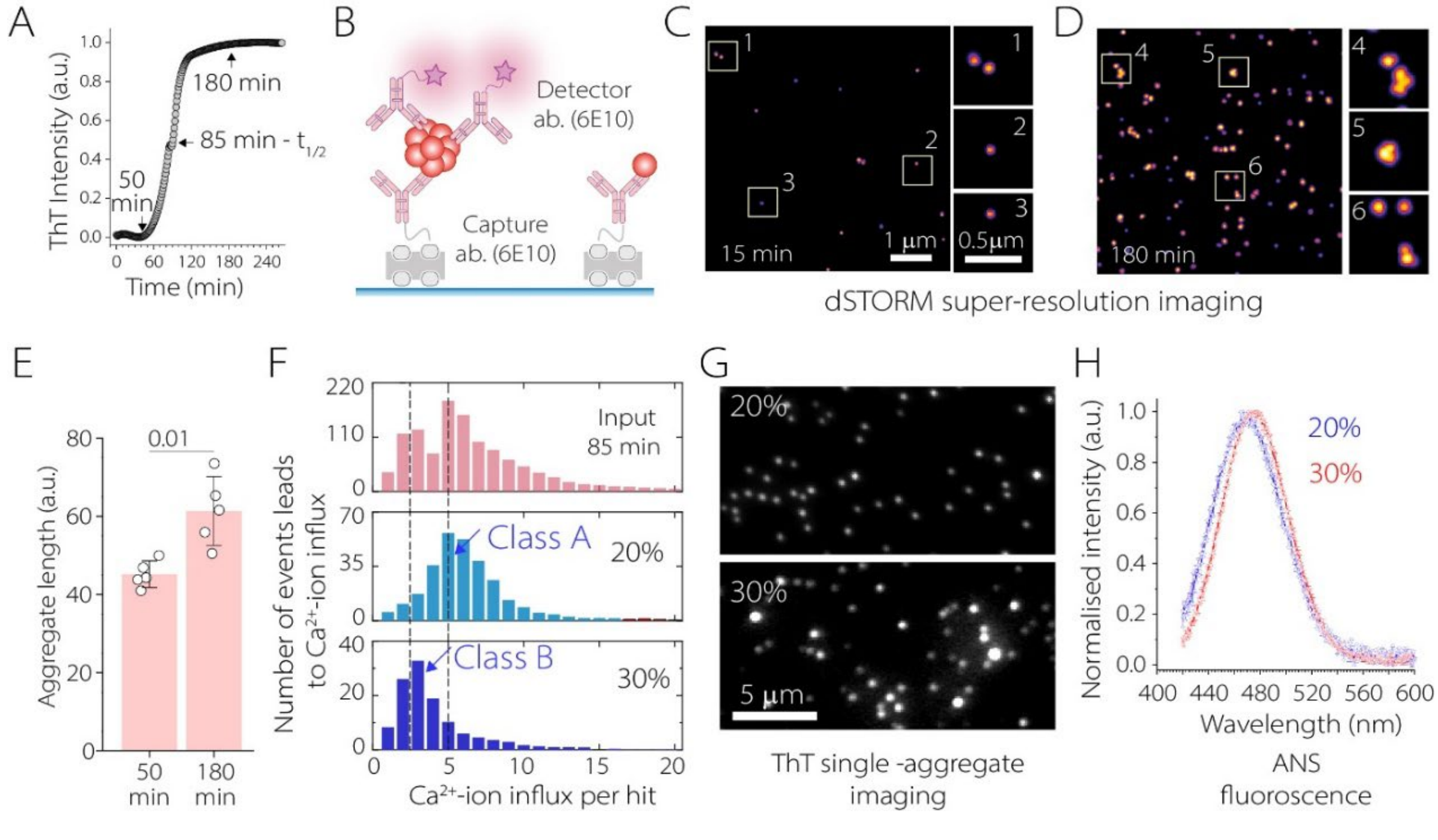

Figure 1. **(A)** ThT aggregation of 3μM of Aβ42 **(B)** Schematic of the single-aggregate imaging assay. Aβ aggregates are captured using biotinylated 6E10antibody and detected using Alexa Fluor 647 labeled 6E10 antibody, enabling imaging of aggregates only, but not unaggregated monomers. **(C-D)** Representative dSTORM images of Aβ aggregates using samples collected at 15 and 180 min during aggregation. Insets show magnified examples. **(E)** Quantification of aggregate size measured from super-resolution imaging. **(F)** Ca^{2+} influx distributions measured for unfractionated aggregates collect at the half-time of the aggregation (top) and for aggregates isolated from 10-20% and 30-40% sucrose-gradient fractions. **(G)** Representative images of individual Aβ42 aggregates present in the 10-20% and

30-40% fractions. **(H)** Normalized ANS fluorescence spectra of the 10-20% and 30-40% sucrose-gradient fractions.

2. To my knowledge, the rationale to use the half-time of the ThT fluorescence signals to normalize different Aβ aggregate samples is to have the same level of cross-β interactions. It holds true if there are only minor Aβ1-42 concentration variations (between 2 μM and 5 μM) because the authors could presume that there was no or little influence on both off-pathway and on-pathway aggregation dynamics. However, after adding 10% and 100% molecular equivalents of BRICHOS R221E, the on-pathway and off-pathway aggregation dynamics could no longer be presumed to be influenced insignificantly even at the same Aβ1-42 concentration (3 μM). In Figure 2D, no BRICHOS R221E-treated sample was analyzed at 85 minutes while 10% BRICHOS R221E-treated sample was analyzed at 105 minutes and 100% BRICHOS R221E-treated sample was analyzed at 275 minutes. A precondition is needed to carry out data comparison: relative to the no BRICHOS R221E-treated control, both on-pathway and off-pathway aggregation dynamics were only slowed down proportionately upon the BRICHOS R221E addition. An unverified presumption stood here that no significant aggregation pathway modulations and redirections took place. I strongly disagree. When BRICHOS R221E is involved, the authors must rule out its possible influence on the aggregation dynamics before normalizing to the 50% of the ThT fluorescence signals when incubated with 3 μM Aβ1-42. As an alternative option, the authors can choose to use the same time point when the on-pathway and off-pathway dynamics was the only variation. In other words, experimental data cannot be compared in a canonical manner when two variations may be activated.

Reply: The aggregation half-time ($t_{1/2}$) is not used to assume proportional slowing of all aggregation pathways; it is a standard kinetic marker that reports the point at which ~50% of the final ThT-positive fibril mass has formed. It is widely used to compare aggregation rates and the effects of accelerators or inhibitors (***Nature Protocol*** *2016, 11, 252*; ***Nature*** *2022,612,123;* ***Science Adv.*** *2026,12,eaeb2729;* ***Nature Chem. Biol.*** *2024,20,981****; Nature Comm.*** *2019,10,225****; PNAS*** *2026,123,e2532775123*). Similarly, in this study, $t_{1/2}$ is used solely to identify comparable mechanistic stages of the reaction, enabling aggregate populations to be compared at equivalent points along the aggregation trajectory irrespective of differences in aggregation rate.

To remove any ambiguity, we further analysed Aβ42 intermediates across the full aggregation time course in the absence and presence of BRICHOS R221E at 10%, 50%, and 100% molar equivalents. As suggested by the reviewer, aliquots were sampled at identical absolute time points for all conditions, and membrane permeabilization was quantified at each point. This provide independent datasets that does not rely on comparing samples at $t_{1/2}$.

Results from these experiments are in agreement with our previous conclusions and showed that BRICHOS R221E delayed Aβ42 aggregation kinetics (**Fig. 2A**) and reduced membrane-permeabilizing activity (**Fig. 2B,C**) in a dose-dependent manner. Crucially, although BRICHOS delayed aggregation and reduced the overall abundance of membrane-damaging species, the

temporal pattern of these species remained the same across all conditions. With or without BRICHOS, membrane-disrupting activity rose during early aggregation, peaked around the aggregation midpoint ($t_{1/2}$), and gradually declined during the plateau phase as fibrils matured (**Fig. 2D-G**).

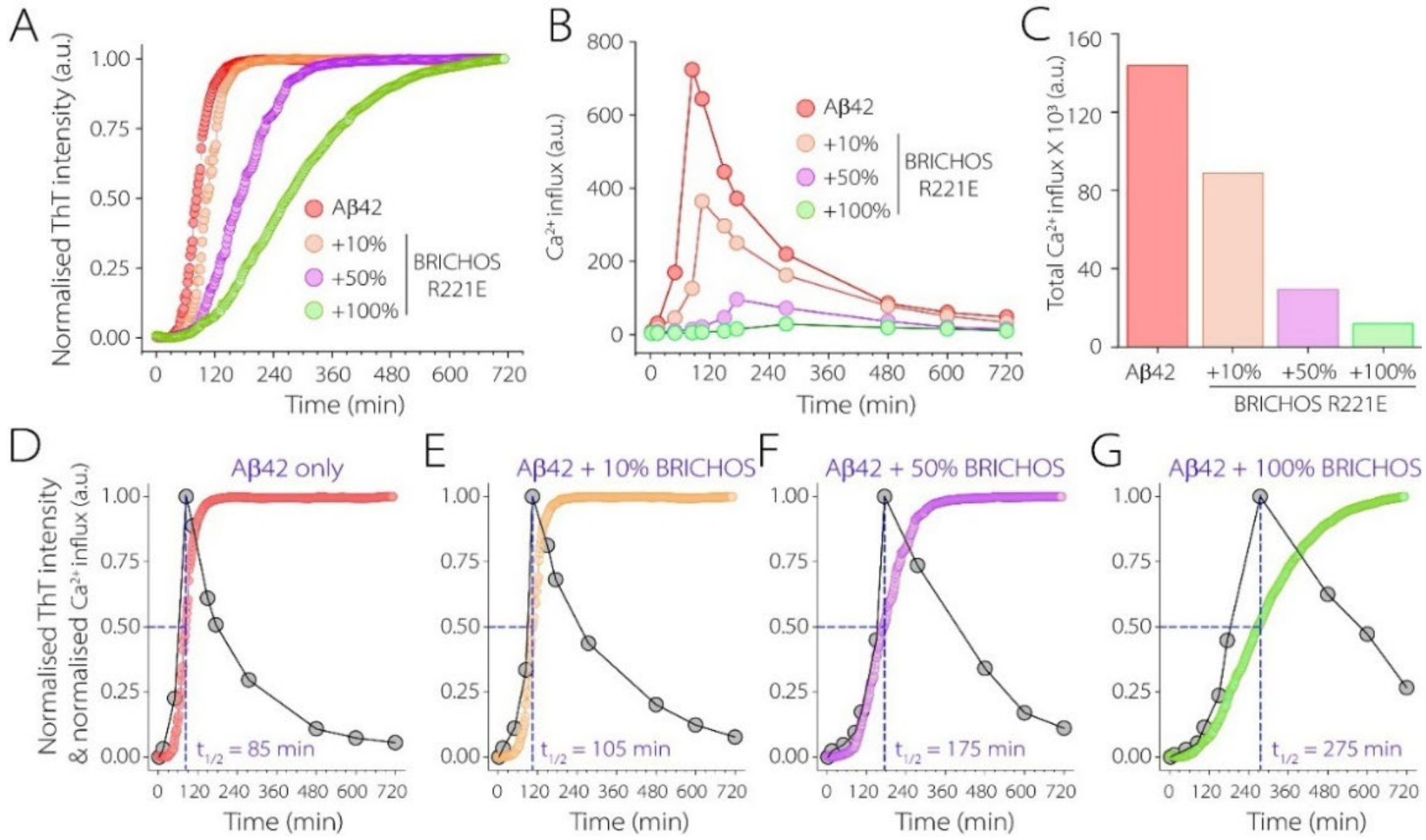

Figure 2. **(A)** Normalized ThT aggregation kinetics of Aβ42 alone or in the presence of increasing BRICHOS R221E concentrations. **(B)** Ca^{2+} influx measured over the same aggregation time course using the vesicle assay. **(C)** Total membrane damage, calculated from the area under the Ca^{2+} influx curve, showing a dose-dependent reduction with increasing BRICHOS R221E concentration **(D-G)** Overlay of normalized ThT aggregation kinetics (coloured) and membrane-damaging activity (grey) for Aβ42 with 0% (D), 10% (E), 50% (F), or 100% (G) BRICHOS R221E. Dashed lines indicate the half-time ($t_{1/2}$) defined as the time at which 50% of the final ThT-positive fibril mass has formed, for each condition. Circles show experimental data points, and lines are included as visual guides.

Therefore, our use of the aggregation mid-point remains a valid and biologically meaningful comparison point. Across all conditions, $t_{1/2}$ corresponds to:

(i) the time at which fibril mass reaches ~50% of its final value, *and*

(ii) the stage at which membrane-damaging intermediates are maximally enriched.

This establishes that half-time ($t_{1/2}$) reflects a comparable mechanistic state even when aggregation kinetics are altered. We have added this data to revised supplementary information as Fig S11 and revised the manuscript accordingly (Line 200-203) .

3. The authors stated that BRICHOS R221E was a chaperone known to slow down aggregation by specifically inhibiting secondary nucleation. In contrast, as the authors discussed on page 17 and showed in Figure 8, both Class A and Class B arised predominantly from primary nucleation. These results seem to stand against each other. If BRICHOS R221E specifically inhibits secondary nucleation, the experiment results should show that only Class B is inhibited in the late aggregation time while no or little inhibiting effects should be observed in the lag phase where the primary nucleation predominates. The experimental results in this

work show that BRICHOS R221E can inhibit both Class A and Class B species. Being faithful to the results, I would rather deduce that the peptide-peptide interface(s), where BRICHOS R221E can act, may play a more important role in the formation of Class A species than that of Class B species. This also echoes well to the sucrose gradient fraction analysis: "lower-density fractions were enriched in class A species, whereas higher-density fractions were enriched in class B aggregates." This hints that the authors may need to characterize the conformational differences between Class A and Class B in case the two species showed similar chemical compositions. I recommend that the authors could use known small-molecule amyloid inhibitors rather than BRICHOS R221E to explore the compositional and conformational differences between these two kinetic intermediates.

Reply: We would like to clarify the interpretation of our data. We did not state, either on page 17 or in the caption of Fig. 8, that both Class A and Class B arise predominantly from primary nucleation. Rather, we stated in those manuscript locations that Class A and Class B have distinct kinetic origins. Class A appears early during the lag phase, when fibril mass is minimal, whereas Class B follows fibril growth and is linked to fibril-dependent secondary nucleation.

Moreover, BRICHOS is known to inhibit fibril formation by suppressing secondary nucleation through binding to fibril surfaces (***Nat. Struct. Mol. Biol.*** *2015, 22, 207;* ***J. Biol. Chem.*** *2026, 302, 113117*). However, this does not necessarily mean that BRICHOS can only interact with fibril surfaces, or that it cannot modulate the activity of non-fibrillar Aβ species. In our manuscript, Class A intermediates are interpreted as predominantly off-pathway to fibril formation. Therefore, the observation that BRICHOS suppresses Class A-mediated membrane damage is not inconsistent with its established effect on fibril formation through secondary nucleation. Rather, our data suggest that BRICHOS affects Class A and Class B through distinct mechanisms: it reduces Class B formation during aggregation by inhibiting fibril-surface-mediated secondary nucleation, whereas it suppresses the membrane-damaging activity of pre-formed Class A species. To further test this interpretation experimentally, we performed additional measurements to distinguish whether Class A and Class B are differentially affected by perturbing secondary nucleation and by adding BRICHOS to pre-formed aggregates.

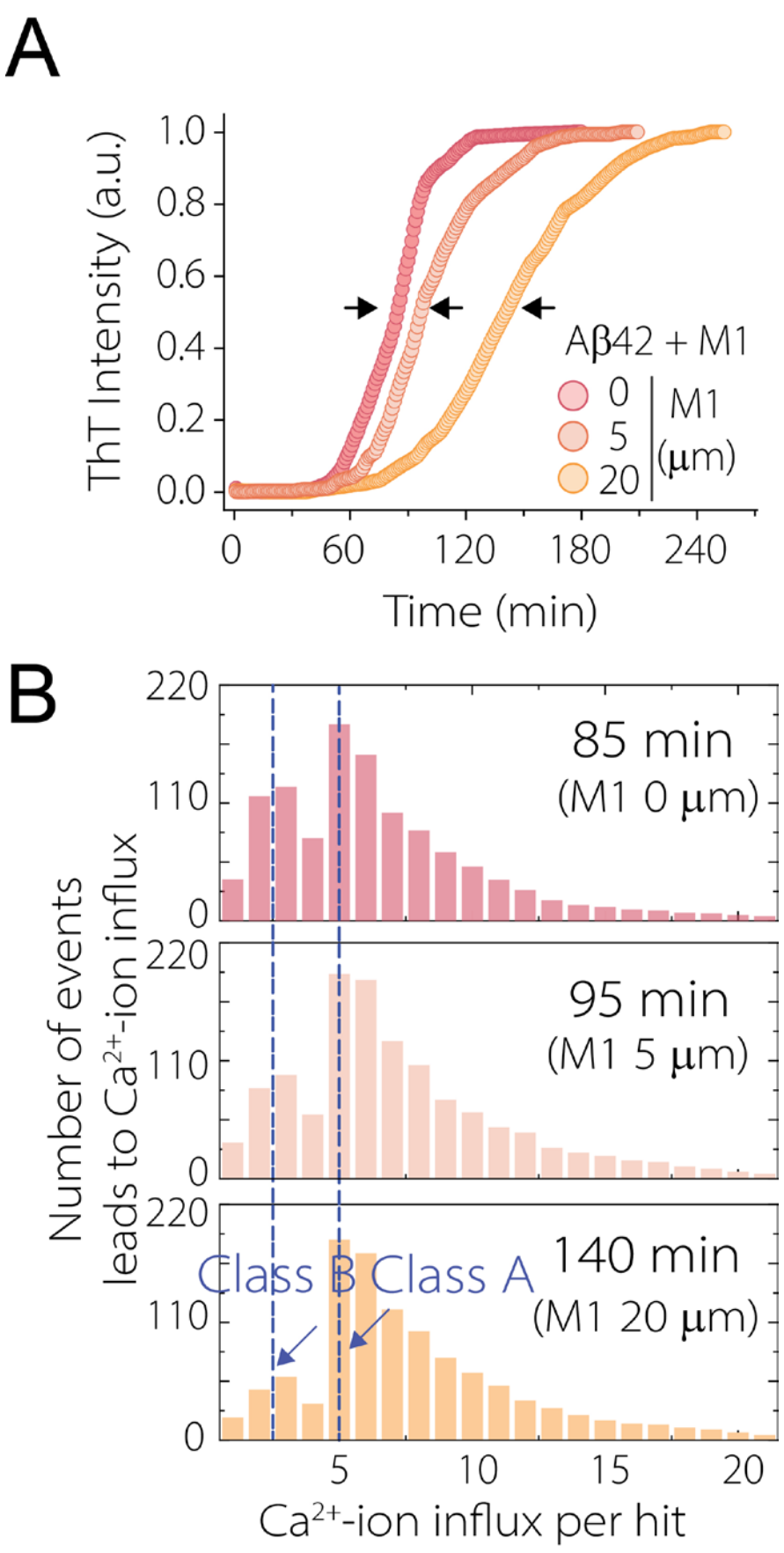

Fig.3 (A) ThT aggregation of Aβ42 in the absence or presence of increasing M1. Arrows indicate the aggregation midpoints analysed. **(B)** Ca^{2+} influx per hit distributions measured at the corresponding midpoints for each aggregation.

First, we tested the small-molecule modulator M1, as suggested by the reviewer, which has shown to inhibit Aβ42 secondary nucleation (***Mol. Syst. Biol.*** *2025,22,69*) (**Fig. 3A**). When Ca^{2+} influx was measured

at the corresponding aggregation midpoints, M1 selectively reduced the Class B population, whereas Class A species remained the dominant membrane-damaging species (**Fig. 3B**). These data support that Class A and Class B are mechanistically distinct intermediates and indicate that Class B, but not Class A, is primarily generated through secondary nucleation.

Second, we incubated pre-formed aggregates collected at the 85-min midpoint with BRICHOS R221E. BRICHOS strongly reduced Class A-mediated Ca^{2+} influx, whereas it had only a minor effect on Class B species (**Fig. 4**). These data suggest that BRICHOS can suppress the membrane-damaging activity of pre-formed Class A species, whereas its effect on Class B occurs mainly during aggregate formation.

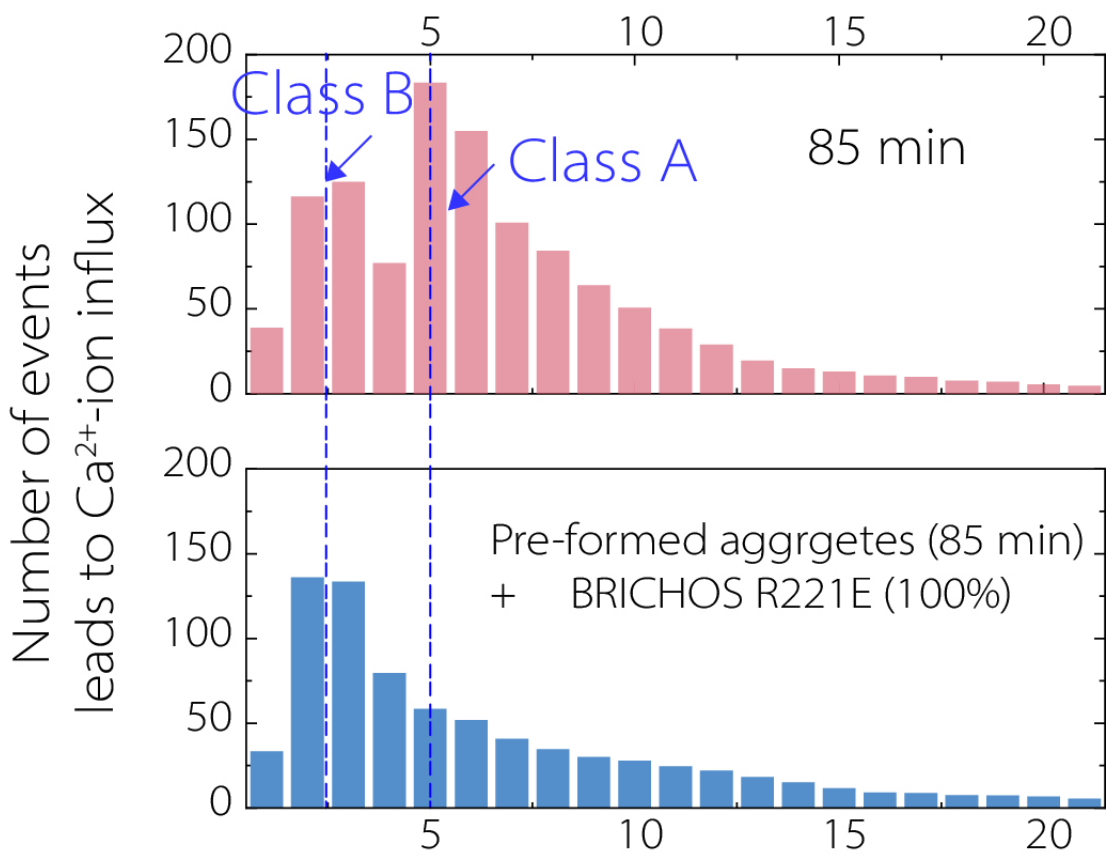

Fig.4 Ca^{2+} influx per hit distributions for Aβ42 collected at the aggregation midpoint (85 min), before and after incubation with 100% molar equivalent BRICHOS.

Third, to independently test which class is linked to secondary nucleation, we performed seeded aggregation using 2.5% pre-formed Aβ42 fibrillar seeds to bypass primary nucleation (***Nat. Comm.*** *2026,17,1933*). Under these conditions, the lag phase was largely abolished (**Fig. 5A**), consistent with minimal contribution from primary nucleation. When Ca^{2+} influx was measured at both early (**Fig. 5B**) and midpoint stages (**Fig. 5C**) of the seeded aggregation, Class B species were strongly enriched, whereas Class A species were much less prominent. In contrast, at comparable stages of unseeded aggregation, both Class A and Class B species were present. These data further support that Class B, but not Class A, is primarily linked to fibril-dependent, on-pathway secondary nucleation.

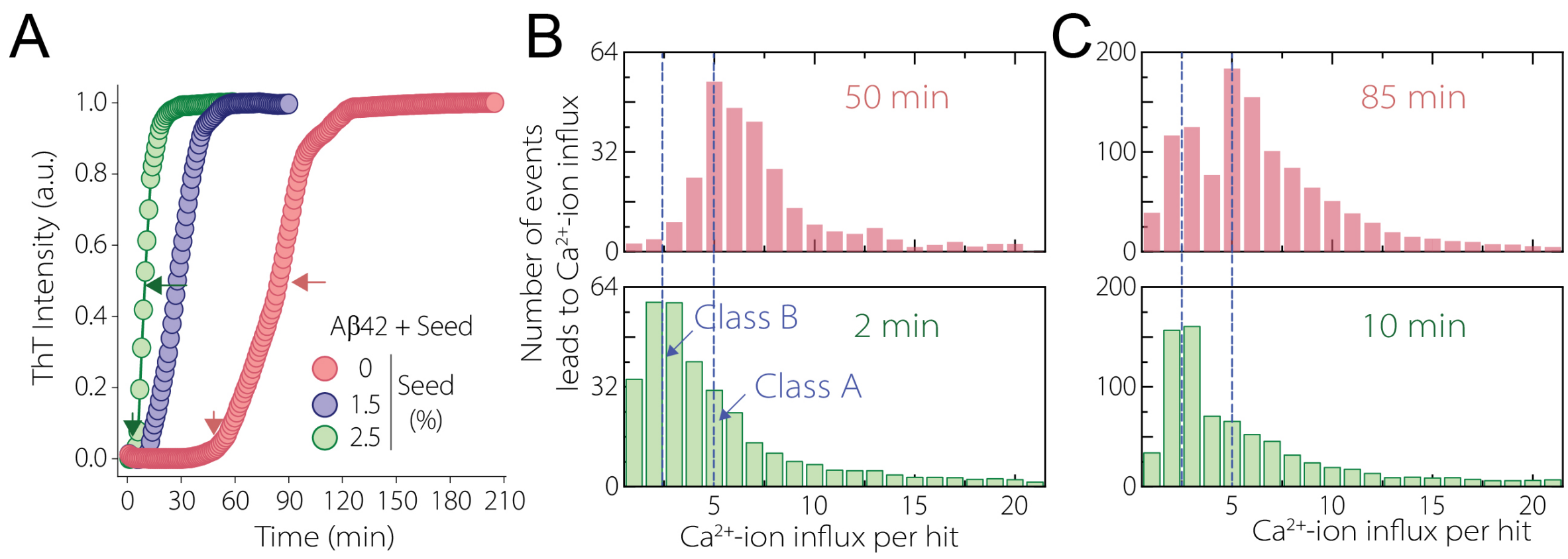

Fig.5 (A) ThT aggregation of Aβ42 in the absence or presence of 1.5% or 2.5% pre-formed fibril seeds. Arrows indicate the time points analysed. **(B-C)** Ca^{2+} influx per hit distributions for unseeded (pink) and 2.5% seeded Aβ42 (green(aggregation at early time points and aggregation midpoints.

Together, these data clarify that Class A and Class B arise through distinct mechanisms. BRICHOS reduces Class B formation by binding to fibril surfaces and blocking secondary nucleation, whereas its effect on Class A is primarily through suppressing the membrane-damaging activity of pre-formed species. These findings support that Class A and Class B are

mechanistically distinct intermediates, with Class B more closely linked to fibril-dependent secondary nucleation. We have revised the manuscript to clarify this distinction (Line 482-485, Line 496-500) and added in Supplementary information as Fig S13,S12 and S14 respectively to better explain how these two classes differ mechanistically.

4. The authors extracted diffusible Aβ1-42 aggregates from the PM frontal cortex tissue of three AD patients. This means that these diffusible Aβ1-42 aggregates by large derives from the amyloid plaques. However, the authors need to be aware that these diffusible Aβ1-42 aggregates may have been chemically engineered upon the amyloid plaque formation. For example, cross-linked amyloid-β dimers have been reported to neurotoxic species such as the Asp1-Glu22 link (Brinkmalm et al., Brain 2019, 142, 1441).

Reply: We agree that Aβ species extracted from postmortem AD brain tissue may include modified aggregates. In the manuscript, we examined pyroglutamate-modified Aβ beginning at position 3, AβpE3-42, one of the common post-translationally modified Aβ species found in AD pathology. We found that AβpE3-42 also generated Class A and Class B intermediates with membrane-permeabilizing activities. We acknowledge that our assay may not capture every toxic Aβ species present in human brain extracts. However, the aim of this study was not to catalogue all toxic Aβ species in the AD brain. Rather, our objective was to establish a functional correspondence between recombinant Aβ42 intermediates and the membrane-disruptive species detected in AD brain extracts, and to determine whether these species map onto distinct aggregation routes.

Minor comments:

5. In Figure 4 and Figure 8, the on-pathway and off-pathway aggregations should be noted as reversible processes.

Reply: We thank the reviewer for this key suggestion. We have now revised Figures 4 and 8 to indicate that the on-pathway and off-pathway aggregation steps are reversible processes.

6. The legend to Figure 4 is a bit messy.

Reply: We thank the reviewer for pointing out this inconsistency in our manuscript. We apologise for the oversight. We have now revised the Figure 4 captions in the main manuscript to ensure that the figure caption matches the figure panels (Line 275-282).

Reviewer #4 (Remarks to the Author):

The authors have gone to great lengths to address the concerns presented to them from all reviewers.

The data that has now been included in the manuscript and the SI adds strength to the study and removes doubt that aggregates were subject to membrane-associated, calcium buffer or dilution effects. The addition of the control experiment assessing BRICHOS affect on vesicle integrity in the absence of Abeta also strengthens the manuscript. The quantification of aggregates in the different brain regions adds clarity for the reader. The use of additional

orthogonal biophysical characterization techniques is also beneficial to get a sense of the biophysical properties of these Class A and Class B aggregates.

In light of these additional data and modifications to the manuscript, I recommend this paper for publication with no further comments.

Reply: We thank the reviewer for their positive assessment of the revised manuscript. We are very pleased that the additional experiments and revisions have addressed the reviewer's concerns and strengthened the study.

Reviewer #5 (Remarks to the Author):

Urbanek et al perform a series of studies to show the correlation of two functionally distinct amyloid-beta (Ab) intermediates with the pathology of Alzheimer's disease.

Study 1: A single-vesicle assay is developed to quantify the membrane-disrupting activity of Ab42 aggregates. Specifically, the nanoscale vesicles composed of neuronal membrane-mimicking lipids were loaded with a dextran-conjugated Ca2+-sensitive dye (Cal-520). The vesicles were then immobilized in a Ca2+-containing buffer on a coverslip and exposed to Ab42 aggregates obtained at different time points in the aggregation kinetics (based on the ThT time trace). The disruption of vesicle membrane was measured by recording Ca2+ influx events. The two peaks in the distribution of these Ca2+ influx events are assumed to associate with two different classes of Ab42 intermediates: Class A intermediates with Ca2+ influx distribution peaking at ~ 5 Ca2+ ions per event and Class B intermediates with Ca2+ influx distribution peaking at ~ 2-3 Ca2+ ions per event. In the same set of experiments, the authors show that BRICHOS R221E (a chaperone known to slow down aggregation by specifically inhibiting secondary nucleation), with concentration equal to that of the Ab42 monomers, completely eliminated the Ca2+ influx events corresponding to Class A events, where as Ca2+ events associated with Class B intermediates remained detectable. They further showed that Ab42 significantly impaired neurite outgrowth in hPSC cortical neurons, which was restored to almost control levels when treated with BRICHOS R221E having the same concentration as that of Ab42.

Reply: We thank the reviewer for their thorough and constructive evaluation of our manuscript. We have addressed all points raised below and have revised the manuscript accordingly. We believe these changes further strengthen the work.

Considering that the classification is based on the Ca2+ influx (functional) and not the extent of physical disruption of the membrane, this set of experiments support the existence of two predominant classes of functional Ab42 intermediates as claimed. I have only one minor concern about this study. No attempt has been made to explain or hypothesize the potential mechanism for the membrane disruption. Based on Figure 1I and 1J, the first Ca2+ influx event seems to occur in the first 1-3 minutes of exposing the vesicles to Ab42 intermediates. What would be the possible mechanism for such rapid membrane disruption?

Reply: We thank the reviewer for this insightful comment. We agree that the precise molecular mechanism underlying the rapid membrane disruption remains to be fully defined. The rapid

onset of Ca^{2+} influx after aggregate exposure is consistent with direct interaction of Aβ42 aggregates with the vesicle membrane, leading to transient membrane permeabilization, possibly through pore-like defects. Our newly added measurements of the hydrophobicity of the two different classes of aggregates also support this hypothesis. We have now added a line to the Discussion to clarify this point (Line 459-461).

Study 2: The next set of experiments show that the two distinct Ab42 intermediate classes have distinct kinetics signatures and damaging potential. This is shown by exposing the vesicles to Ab42 aggregates obtained at different time points along the aggregation kinetics (based of ThT signal) and plotting the distribution Ca2+ influx events. At earlier time, these distributions show a dominant peak corresponding to Class A events, which slowly decays with the time along the aggregation kinetics. The peak corresponding to Class B events, on the other hand, becomes pronounced and persists for the remaining of aggregation kinetics. The second part of this study uses a kinetics model to globally fit the ThT traces and show that the model results in a better fit when Class A intermediates are considered to grow along the off-pathway and Class B intermediates are considered to grow along the on-pathway (seeds for the fibrils).

The experiments presented support the claim that the two distinct Ab42 intermediate classes have different damaging potentials, with Class A intermediates more toxic than Class B intermediates. However, the claim about the distinct kinetics has a caveat that needs to be acknowledged. After transferring the intermediates to the vesicles, the aggregate species are assumed to keep the same kinetic state (e.g. species that were off-pathway in the solution remain off-pathway after getting transferred to the vesicles) for the duration of 20 min (or longer) experiment.

Reply: We thank the reviewer for this important point. We agree that, after transfer to vesicles, some intermediates may continue to evolve during dilution or be lost during the assay, and therefore the measured species may not perfectly represent every aggregate present at the original sampling time point. We have now acknowledged this limitation in the Discussion (Line 570-572).

Function 2F1 in equation 2 is not defined.

Reply: We apologise for the oversight. The ${}_2F_1[a,b;c;z]$ is the Gaussian hypergeometric function. We have added this information in the method section of the manuscript (Line 832).

Study 3: Based on immunohistochemistry of frontal cortex from three AD cases, this set of experiments show that human AD brain contains two classes of toxic Ab42 intermediates, and extracts from these AD-affected brains impair neurite outgrowth in hPSC-derived cortical neurons. The conclusions drawn from these experiments are well-supported by the data.

Study 4: This set of experiments show that BRICHOS R221E suppress the formation of Ab42 oligomeric intermediates and attenuates neuronal and astrocytic dysfunction. This includes restoration of neurite outgrowth in neurons and reduction in pro-inflammatory cytokines. The conclusions drawn from these experiments are well-supported by the data.

Study 5: The two toxic Ab42 intermediates are also detected in a mouse model of AD, and BRICHOS R221E specifically suppress Class A intermediates in this mouse model. The conclusions drawn from these experiments are well-supported by the data.

Reply: We are pleased that the reviewer finds the conclusions well supported by the data.

General comments:

1. Functional classification without direct structural characterization: While the authors classify the two intermediate classes based on their Ca^{2+} influx signatures, the structural basis for this functional distinction remains indirect. The sucrose gradient fractionation and dSTORM imaging data (Fig. S7) provide supporting evidence for physical distinctness between Class A and Class B species, but a more direct structural characterization — such as native mass spectrometry or cryo-EM of isolated fractions — would strengthen the claim that these functional classes correspond to structurally distinct assemblies. The authors should acknowledge this as a limitation of the current classification approach.

Reply: We agree with the reviewer that direct structural characterization of different classes of intermediates would be valuable and provide important insight into their structure and function. We have now acknowledged this limitation in the Discussion (Line 573-575).

2. Lipid vesicle composition versus native neuronal membranes: The membrane permeabilization assay employs vesicles composed of neuronal membrane-mimicking lipids, which, while carefully chosen, do not fully recapitulate the complexity of native neuronal membranes. Factors such as membrane asymmetry, protein composition, cholesterol content, and membrane tension in living cells could influence the Ca^{2+} influx signatures and the relative membrane-disrupting potency of Class A and Class B intermediates. It would be valuable for the authors to discuss whether the functional classification and the relative potency of the two intermediate classes are expected to hold under more physiologically relevant membrane conditions.

Reply: We agree that our vesicle-based reductionist system does not capture the full complexity of native neuronal membranes. Its purpose here is to provide a controlled and reproducible platform for comparing the membrane-disrupting activities of Aβ aggregates at the single aggregate level. Importantly, the same two functional classes of intermediates were detected not only in recombinant Aβ42 preparations, but also in mouse brain tissue and human AD brain extracts, demonstrating that these classes are not artefacts of the simplified experimental system. While the absolute Ca^{2+} influx signatures, and thus the inferred membrane permeabilization values, may differ in native membranes, our data support that the functional distinction between Class A and Class B is biologically relevant. We agree that future studies using more complex membrane systems or intact cellular membranes will be important to determine how native membrane composition modulates their relative potency, and we have now clarified this point in the revised manuscript (Line 213-217).

3. Limited human brain cohort with unexplained inter-individual variability: The demonstration that both Class A and Class B intermediates are present in human Alzheimer's brain is an

important finding; however, it is based on postmortem frontal cortex tissue from only three sporadic AD cases. Furthermore, the relative contributions of Class A and Class B intermediates vary considerably across the three patients (Fig. 5F), yet this variability is not discussed. It remains unclear whether these differences reflect disease stage, rate of progression, individual genetic background, or other factors. The authors should acknowledge the limited sample size as a constraint on the generalizability of their conclusions and discuss possible explanations for the observed inter-individual variability in intermediate class distribution.

Reply: We agree with the reviewer that the human post-mortem brain tissue analysis is based on a limited cohort, and we now explicitly acknowledge this constraint in the Discussion (Line 532-534). The observed inter-individual variation in Class A and Class B distribution may reflect several factors, including disease stage, progression rate, genetic background, postmortem interval, or regional pathology burden. This variability highlights the biological heterogeneity of AD and underscores the need for larger studies. Crucially, the platform we establish here provides a means to investigate how disease-associated variables influence the distribution of toxic Aβ oligomeric intermediate populations across broader patient cohorts. We have explicitly addressed this point in the Discussion, where we clarify the interpretation of the human brain tissue results (Line 522-526, Line 532-534).

4. Kinetic modeling assumptions underlying the on- and off-pathway classification: The assignment of Class A intermediates as off-pathway and Class B intermediates as on-pathway to fibril formation rests substantially on global kinetic modeling rather than direct experimental demonstration of interconversion or incorporation into fibrils. While the modeling approach is rigorous and the differential sensitivity to BRICHOS R221E provides complementary support, the conclusions are inherently dependent on the assumptions built into the kinetic framework — in particular, the reaction orders and the relative contributions of primary versus secondary nucleation. The authors should more explicitly discuss the limitations of this modeling approach and clarify how sensitive their pathway assignments are to variations in these underlying assumptions.

Reply: We thank the reviewer for this important point. We note that the relative contributions of primary and secondary nucleation, and the reaction orders, are not assumptions; they are determined directly from fits to the experimental aggregation data, and we have now clarified this in the manuscript. Moreover, our pathway assignments do not rely on modelling alone. Class A appears early during the lag phase, when fibril mass is minimal, consistent with a dominant contribution from primary nucleation. In contrast, Class B emerges during fibril growth, is selectively reduced by inhibition of secondary nucleation, and is strongly enriched when primary nucleation is bypassed by seeding. Thus, the distinction between on- and off-pathway behaviour is supported by convergent kinetic, inhibitor, and seeding evidence, not solely by the modelling framework. We have now expanded the Discussion to clarify how these conclusions are reached (Line 469-520) and explicitly acknowledge the limitations inherent to kinetic modelling (Line 513-516).

Overall: I find this study very interesting and significant for further progress in the field. The authors have done a very good job in responding to the previous round of reviews. I recommend the publication of this paper in Nature Communications after the concerns raised above are addressed.

Reply: We are pleased that the reviewer finds the study interesting. We believe that our responses adequately address the remaining concerns and thank the reviewer for recommending the manuscript for publication.
